# Supplementary material for: Selecting appropriate variables for detecting grassland to cropland changes using high resolution satellite data
Source: PeerJ. 2018 Sep 6;6:e5487. doi: 10.7717/peerj.5487 (PMC6129385; doi:10.7717/peerj.5487)
Supplement: Supplemental Information 2 — The value of AIC specifies the information potential of the model. Models verified in the study by object-based classification are highlighted in grey. [file peerj-06-5487-s002.docx]

| **No. of variables** | **Change detection model** | **AIC^1^** |
| --- | --- | --- |
| **One** | Normalized Difference Vegetation Index | **5633.39** |
| **Two** | Sum Green Index, Wetness | **4887.197** |
| **Three** | Normalized Difference Vegetation Index, Wetness, Fifth | **4592.41** |
| **Four** | Sum Green Index, PCA2, PCA4, Wetness | **4362.041** |
| **Five** | Normalized Difference Vegetation Index, Wetness, Fifth, Brightness, Sum Green Index | **4263.74** |
| **Six** | Normalized Difference Vegetation Index, Sum Green Index, Brightness, Fifth, PCA2, Wetness | **4090.38** |
| **Seven** | Normalized Difference Vegetation Index, Wetness, Fifth, Brightness, Sum Green Index, Second Moment, PCA 2 | **4060.35** |
| **Eight** | Normalized Difference Vegetation Index, Wetness, Fifth, Brightness, Sum Green Index, Second Moment, PCA 2, PCA 1 | **4036.42** |
| **Nine** | Normalized Difference Vegetation Index, Wetness, Fifth, Brightness, Sum Green Index, Second Moment, PCA 2, PCA 1, PCA 3 | **4011.35** |
| **Ten** | Normalized Difference Vegetation Index, Wetness, Fifth, Brightness, Sum Green Index, Second Moment, PCA 2, PCA 1, PCA 3, PCA 4 | **3990.64** |
| **Eleven** | Normalized Difference Vegetation Index, Wetness, Fifth, Brightness, Sum Green Index, Second Moment, PCA 2, PCA 1, PCA 3, PCA 4, PCA 7 | **3977.46** |
| **Twelve** | Normalized Difference Vegetation Index, Wetness, Fifth, Brightness, Sum Green Index, Second Moment, PCA 2, PCA 1, PCA 3, PCA 4, PCA 7, Data Range | **3961.40** |
| **Thirteen** | Normalized Difference Vegetation Index, Wetness, Fifth, Brightness, Sum Green Index, Second Moment, PCA 2, PCA 1, PCA 3, PCA 4, PCA 7, Data Range, Contrast | **3954.82** |
| **Fourteen** | Normalized Difference Vegetation Index, Wetness, Fifth, Brightness, Sum Green Index, Second Moment, PCA 2, PCA 1, PCA 3, PCA 4, PCA 7, Data Range, Contrast, Skewness | **3950.90** |

^1^AIC (Akaike Information Criterion)
