# Supplementary material for: Selecting appropriate variables for detecting grassland to cropland changes using high resolution satellite data
Source: PeerJ. 2018 Sep 6;6:e5487. doi: 10.7717/peerj.5487 (PMC6129385; doi:10.7717/peerj.5487)
Supplement: Supplemental Information 3 — PA, Producer’s accuracy; UA, User’s accuracy; O, Omission and C, Commission. Change, No change and Sum values represent number of objects. PA, UA, O and C are in percent (%). [file peerj-06-5487-s003.docx]

Model based of **on**e variable

|  | **Change** | **No Change** | **Summary** | **PA (%)** | **UA (%)** | **O (%)** | **C (%)** |
| --- | --- | --- | --- | --- | --- | --- | --- |
| **Change** | 92 | 11 | 103 | 46.00 | 89.32 | 54.00 | 10.68 |
| **No Change** | 108 | 789 | 897 | 98.63 | 87.96 | 1.38 | 12.04 |
| **Summary** | 200 | 800 | 1000 | **Overall accuracy 88.10 %, Kappa 0.5455** | | | |

Model based of **three** variables

|  | **Change** | **No Change** | **Summary** | **PA (%)** | **UA (%)** | **O (%)** | **C (%)** |
| --- | --- | --- | --- | --- | --- | --- | --- |
| **Change** | 99 | 9 | 108 | 49.50 | 91.67 | 50.50 | 8.33 |
| **No Change** | 101 | 791 | 892 | 98.88 | 88.68 | 1.13 | 11.32 |
| **Summary** | 200 | 800 | 1000 | **Overall accuracy 89.00 %, Kappa 0.5846** | | | |

Model based of **five** variables

|  | **Change** | **No Change** | **Summary** | **PA (%)** | **UA (%)** | **O (%)** | **C (%)** |
| --- | --- | --- | --- | --- | --- | --- | --- |
| **Change** | 93 | 8 | 101 | 46.50 | 92.08 | 53.50 | 7.92 |
| **No Change** | 107 | 792 | 899 | 99.00 | 88.10 | 1.00 | 11.90 |
| **Summary** | 200 | 800 | 1000 | **Overall accuracy 88.50 %, Kappa 0.5587** | | | |

Model based of **seven** variables

|  | **Change** | **No Change** | **Summary** | **PA (%)** | **UA (%)** | **O (%)** | **C (%)** |
| --- | --- | --- | --- | --- | --- | --- | --- |
| **Change** | 104 | 14 | 118 | 52.00 | 88.14 | 48.00 | 11.86 |
| **No Change** | 96 | 786 | 882 | 98.25 | 89.12 | 1.75 | 10.88 |
| **Summary** | 200 | 800 | 1000 | **Overall accuracy 89.00 %, Kappa 0.5938** | | | |

Model based of **fourteen** variables

|  | **Change** | **No Change** | **Summary** | **PA (%)** | **UA (%)** | **O (%)** | **C (%)** |
| --- | --- | --- | --- | --- | --- | --- | --- |
| **Change** | 111 | 13 | 124 | 55.50 | 89.52 | 44.50 | 10.48 |
| **No Change** | 89 | 787 | 876 | 98.38 | 89.84 | 1.63 | 10.16 |
| **Summary** | 200 | 800 | 1000 | **Overall accuracy 89.80 %, Kappa 0.6283** | | | |

Model based of **Landsat image**

|  | **Change** | **No Change** | **Summary** | **PA (%)** | **UA (%)** | **O (%)** | **C (%)** |
| --- | --- | --- | --- | --- | --- | --- | --- |
| **Change** | 118 | 14 | 132 | 59.00 | 89.39 | 41.00 | 10.61 |
| **No Change** | 82 | 786 | 868 | 98.25 | 90.55 | 1.75 | 9.45 |
| **Summary** | 200 | 800 | 1000 | **Overall accuracy 90.40 %, Kappa 0.6562** | | | |
